# Differences in both prevalence and titre of specific immunoglobulin E among children with asthma in affluent and poor communities within a large town in Ghana

**DOI:** 10.1111/j.1365-2222.2011.03832.x

**Published:** 2011-11

**Authors:** W Stevens, E Addo-Yobo, J Roper, A Woodcock, H James, T Platts-Mills, A Custovic

**Affiliations:** 1 University of Virginia Asthma and Allergic Diseases CenterCharlottesville, VA, USA; 2 Department of Child Health, Komfo Anokye Teaching HospitalGhana; 3 The University of Manchester, Manchester Academic Health Science Centre, NIHR Translational Research Facility in Respiratory Medicine, University Hospital of South Manchester NHS Foundation TrustManchester, UK

**Keywords:** Africa, asthma, body mass index, exercise-induced bronchospasm, specific IgE, total IgE

## Abstract

**Background:**

Reports from several African countries have noted an increasing prevalence of asthma in areas of extensive urbanization.

**Objective:**

To investigate the relevance of allergen-specific sensitization and body mass index (BMI) to asthma/wheezing and exercise-induced bronchospasm (EIB) among children from affluent and poorer communities within a large town in Ghana.

**Methods:**

Children with physician-diagnosed asthma and/or current wheezing aged 9–16 years (*n*=99; cases) from three schools with differing socio-economic backgrounds [urban affluent (UA), urban poor (UP) or suburban/rural (SR)] were recruited from a cross-sectional study (*n*=1848) in Kumasi, Ghana, and matched according to age, sex and area of residence with non-asthmatic/non-wheezy controls. We assayed sera for IgE antibodies to mite, cat, dog, cockroach, *Ascaris* and galactose-α-1,3-galactose.

**Results:**

Children from the UA school had the lowest total serum IgE. However, cases from the UA school had a higher prevalence and mean titre of sIgE to mite (71.4%, 21.2 IU/mL) when compared with controls (14.3%, 0.8 IU/mL) or cases from UP (30%, 0.8 IU/mL) and SR community (47.8%, 1.6 IU/mL). While similar findings were observed with EIB in the whole population, among cases there was no difference in IgE antibody prevalence or titre between children with or without EIB. BMI was higher among UA children with and without asthma; in UP and SR communities, children with EIB (*n*=14) had a significantly higher BMI compared with children with asthma/wheezing without EIB (*n*=38) (18.2 vs. 16.4, respectively, *P*<0.01).

**Conclusions and Clinical Relevance:**

In the relatively affluent school, asthma/wheezing and EIB were associated with high titre IgE antibodies to mite, decreased total IgE, and increased BMI. This contrasted with children in the urban poor school and suggests that changes relevant to a Western model of childhood asthma can occur within a short geographical distance within a large city in Africa.

*Cite this as*: W. Stevens, E. Addo-Yobo, J. Roper, A. Woodcock, H. James, T. Platts-Mills and A. Custovic, *Clinical & Experimental Allergy*, 2011 (41) 1587–1594.

## Introduction

The prevalence of asthma and atopy in sub-Saharan Africa has been rising steadily over the past two or three decades, particularly in areas of increased urbanization [1–8]. Indeed, some reports have documented a prevalence of asthma in African cities as high as in westernized nations [9]. In general, this increase in prevalence has not been observed in African villages [1–5]. Thus, the comparison of different populations within African countries provides an opportunity to investigate the factors that are relevant to the development of a Western-style pattern of asthma.

While there is currently no unifying explanation for these observations, several factors have been identified that may be important in influencing the development of asthma in urban rather than rural African communities. Studies over a 10-year period examining school-aged children from Kumasi, Ghana indicated a dramatic increase in the prevalence of exercise-induced bronchospasm (EIB) and allergic skin sensitization [10]. Sensitization to both dust mite and cockroach by skin test were shown to be risk factors for asthma amongst these urban Ghanaian children [11], and the prevalence of airway hyper-reactivity assessed by EIB was markedly higher in children from an urbanized, relatively affluent community compared with children from urban poor or rural communities [1, 10].

In this study, we focused on identifying factors that could help to explain these observations. As both allergen sensitization [11] and obesity [12–14] have been shown to contribute to asthma development in children, we investigated the relevance of allergen-specific sensitization and body mass index (BMI) to asthma among schoolchildren in Kumasi, and whether the associations of asthma and EIB with specific IgE (sIgE) antibody responses and BMI differs between urban affluent, urban poor and suburban/rural children.

## Methods

### Study design

Case-control study in which cases of asthma and non-asthmatic controls were recruited from a large cross-sectional study amongst schoolchildren aged 9–16 in three schools in Kumasi, Ghana [10].

### Setting and sources and methods of case ascertainment and control selection

We conducted a cross-sectional study in 2003 to investigate the prevalence of asthma and atopy [10], as well as any changes in prevalence compared with our survey from 1993 [1]. All children were asked to complete a validated questionnaire [15], underwent exercise challenge, and were skin-prick tested. We classified participants into three different communities based upon attending a school in (i) an urban affluent (UA) community (University of Science and Technology Primary and Junior Secondary School), (ii) an urban poor (UP) community (the Kotei Primary and Junior Secondary Schools) or (iii) a suburban/rural (SR) community (Ohwim Primary and Junior Secondary schools) [1, 10]. Of note, the Ohwim Primary and Junior Secondary school was defined as a rural community in 1993 [1], but due to changes in population size, economic activities and lifestyles in the area over the 10 years to 2003, this community is better designated as suburban/rural [10].

Following the initial cross-sectional survey, in a nested case–control study we recruited children who reported physician-diagnosed asthma and/or history of current wheeze (wheeze within previous 12 months; cases) and matched them according to age, sex and area of residence/school (UA, UP, SR) with children who reported no wheeze or physician diagnosis of asthma (controls). The Ghana Education Service and the management of the University of Science and Technology Primary and Junior Secondary Schools approved this study, and parental consent was obtained through the heads of schools.

### Definitions of variables

#### Exercise-induced bronchospasm

Exercise challenge was carried out as described previously [10]. Briefly, before exercise, resting peak expiratory flow rate (PEFR) was measured. All children were exercised by running outdoors for 6 min with a target heart rate set at >170 b.p.m. or >85% of maximum for age (whichever was higher). Five and 8 min following exercise, PEFR was again measured and the lower of the two readings recorded. Based on previously established criteria [1, 10, 16], EIB was defined as >12.5% reduction in PEFR following exercise challenge.

#### Serum immunoglobulin E antibody assays

Total IgE and specific IgE to mite, cockroach (*Blatella germanica*), cat, dog and *Ascaris* were measured using commercially available ImmunoCAP assays (Phadia US, Portage, MI, USA). Specific IgE to the novel carbohydrate allergen galactose-α-1,3-galactose (α-gal), which is a putative marker for ectoparasite exposure, was measured using a modified ImmunoCAP assay where 5 μg of Cetuximab (ImClone, Branchburg, NJ, USA) was biotinylated and bound to the solid phase of a streptavidin ImmunoCAP (Phadia US) [17, 18]. All serum assays were run on the ImmunoCAP 250 instrument at the University of Virginia in a blinded manner. Results were expressed as IU/mL with 1 IU equivalent to approximately 2.4 ng and the limit of detection being 0.35 IU/mL.

#### Helminth infection

The presence of parasites in stool specimens was determined on a wet film (iodine preparation), and more detailed analysis of the concentration of cysts and eggs was performed with formol-ether sedimentation [19]. The Stoll's method was used for the quantitative estimation of ova in faeces [20].

### Statistical analysis

Statistical analysis was carried out using SPSS 15.0 (SPSS Inc., Chicago, IL, USA), Stata 6.0 (Stata Corp, College Station, TX, USA) and GraphPad Prism (GraphPad Software, San Diego, CA, USA). Total IgE and BMI data followed a log-normal distribution; the data are presented as geometric means (GM) and 95% confidence interval (CI). Following univariate analysis, IgE responses which were found to be associated with asthma group were further examined in a multivariate regression analysis with adjustment for BMI, maternal asthma, type of fuel used for cooking and tobacco smoke exposure. The size of the effect was measured by using the odds ratios (ORs) and 95% CIs.

Comparisons between the communities were carried out using anova, with *post hoc* test (Tukey's) if necessary. We used a χ^2^-test and Fisher's exact test for comparisons between the categorical variables and Mann–Whitney *U*-test to compare the titres of specific IgE between different groups.

#### Power calculation

Sample size of 180 children (90 cases and 90 controls) gives >80% power at 5% significance to detect an odds ratio of 3.0 associated with binary factors of 20–60% prevalence.

## Results

### Participant flow and demographics

Of 1848 children who participated in the initial cross-sectional study [10], 1152 returned completed questionnaires. A total of 126 children reported current wheezing (39 UA, 55 UP, 32 SR), 40 had physician-diagnosed asthma (18 UA, 15 UP, 7 SR), 67 have consulted their physician for wheeze (32 UA, 22 UP, 13 SR) and 57 received asthma medication (23 UA, 19 UP, 15 SR). Of those, 99 children with a physician-diagnosed asthma and/or a history of current wheeze agreed to take part in the current study (37 UA, 36 UP and 26 SR) and were matched according to age, sex and area of residence/school with asymptomatic non-asthmatic controls. Of 198 enrolled children, 181 provided serum samples for IgE antibody assays and were included in this analysis [70 UA (35 cases), 63 UP (30 cases), 48 SR (23 cases)]. Of those with serum samples, one case and one control did not complete the exercise challenge.

The children from the SR school were somewhat older, but this did not reach statistical significance (*P*=0.15, Table 1). There was a highly significant difference in BMI between the three communities (*P*<0.001, anova), with *post hoc* test (Tukey's) indicating that children from the UA community had significantly higher BMI than either UP (*P*=0.001) or SR children (*P*<0.001) (Table 1).

**Table 1:** Demographics of the study population

	Cases/controls	Cases/controls with EIB	Gender (male/female)	Age, years Mean (95% CI)	Body mass index GM (95% CI)	Total IgE, IU/mL GM (95% CI)
Total*	88/93†	34/1	92/89	11.9 (11.6–12.2)	17.2 (16.8–17.6)	258 (204–326)
Communities						
Urban affluent	35/35	20/1	28/42	11.7 (11.3–12.1)	18.3 (17.6–19.0)‡	166 (110–251)‡
Urban poor	30/33	5/0	34/29	11.7 (11.3–12.1)	16.7 (16.2–17.2)	331 (226–485)
Suburban/rural	23/25	9/0§	30/18	12.3 (11.7–13.0)	16.4 (15.8–17.1)	351 (233–528)

* Of 1848 children who participated in the cross-sectional study, 935 were male (50.6%).

† Of the 99 children with physician-diagnosed asthma (cases), 51 were male (51.5%) while 50.8% of the controls were male.

‡ BMI was significantly higher (*P*<0.01) and total IgE was significantly lower (*P*<0.03) in the urban affluent school.

§ Within the suburban/rural community, one case and one control did not complete the exercise challenge.

CI, confidence interval; BMI, body mass index.

Owing to a shortage of serum, cockroach sIgE was only measured in 179 participants, while IgE to dust mite, α-gal, cat, dog, *Ascaris* and total IgE were measured in all children. Total serum IgE differed significantly between the three communities (*P*=0.01, anova) with the GM (95% CI) of the total IgE among UA children (166 IU/mL, 110–251 IU/mL) being significantly lower compared with children in the UP (331 IU/mL, 226–485 IU/mL; *P*=0.003) or SR school (351 IU/mL, 233–528 IU/mL, *P*=0.03). There was no difference between the UP and SR communities (*P*=0.98) (Table 1).

Parasites were identified in only four stool samples (*Ascaris lumbricoides* in one, *Entamoeba histolytica* in one and hookworm in two).

### Specific immunoglobulin E antibody responses and asthma in the three schools

In the whole population, we observed a markedly higher prevalence of positive sIgE to mite and cockroach amongst cases compared with controls (mite: 51.1% vs. 16.1% *P*<0.001; cockroach: 59.3% vs. 31.2%, *P*<0.001; Table 2). The proportion of children with sIgE to *Ascaris* was also higher among cases (52.3% vs. 36.6%, *P*=0.03). There were no significant differences between the groups for sIgE to cat, dog and α-gal (Table 2). In the multiple logistic regression, positive sIgE to dust mite (OR 4.83, 95% CI 1.89–13.4, *P*=0.001) and cockroach (OR 2.20, 95% CI 1.00–4.93, *P*=0.05) remained the only significant and independent associates of asthma group, with no association found between asthma and sIgE to *Ascaris* (0.84, 0.37–1.92, *P*=0.68). Of note, similar results were obtained in a conditional logistic regression with appropriate adjustment for the one-to-one matching of the groups (positive sIgE to mite, OR 6.80, 95% CI 2.66–17.39, *P*<0.001; cockroach, OR 2.82, 95% CI 1.42–5.61, *P*=0.003).

**Table 2:** Number (proportion) of children in the total population (*n*=181) with detectable IgE to specific allergens and asthma

	Asthma (*n=88)*	No asthma (*n*=*93*)	*P*-value
Mite	45	15	<0.001
	(51.1%)	(16.1%)	
Ascaris	46	34	0.03
	(52.3%)	(36.6%)	
Cockroach	51	29	<0.001
	(59.3%)	(31.2%)	
Cat	3	1	0.36
	(3.4%)	(1.1%)	
Dog	13	7	0.16
	(14.8%)	(7.5%)	
Galactose-α 1,3-galactose	7	6	0.70
	(8.0%)	(6.5%)	

#### Prevalence of immunoglobulin E antibodies

Given the high overall prevalence of IgE antibodies to mite, cockroach and *Ascaris*, we proceeded to analyze both the prevalence and the titres of IgE antibodies in the three communities. The presence of detectable specific IgE antibodies to dust mite and cockroach was significantly associated with asthma in UA and the SR, but not UP children (Table 3). By contrast, IgE antibodies to *Ascaris* were significantly (*P*=0.02) associated with asthma in the UA children only. For IgE antibodies to α-gal, there was no significant association with asthma, but strikingly, these were uniformly negative among the UA children (Table 3).

**Table 3:** The pattern of specific IgE antibody responses, BMI, and asthma in three different schools

	Urban affluent		Urban poor		Suburban/rural	
			
	Asthma Yes (*n*=35)	Asthma No (*n*=35)	Asthma Yes (*n*=30)	Asthma No (*n*=33)	Asthma Yes (*n*=23)	Asthma No (*n*=25)
Mite	25**	5	9	7	11*	3
Frequency (%)	(71.4%)	(14.3%)	(30%)	(21.2%)	(47.8%)	(12%)
Ascaris	14*	5	17	17	15	12
Frequency (%)	(40%)	(14.3%)	(56.7%)	(51.5%)	(65.2%)	(48%)
Cockroach	23**	8	11	12	17*	9
Frequency (%)	(65.7%)	(22.9%)	(36.7%)	(36.4%)	(73.9%)	(36%)
Cat	1	0	0	0	2	1
Frequency (%)	(2.9%)	(0%)	(0%)	(0%)	(8.7%)	(4%)
Dog	5	1	4	4	4	2
Frequency (%)	(14.3%)	(2.9%)	(13.3%)	(12.1%)	(17.4%)	(8%)
α-gal	0	0	3	4	4	2
Frequency (%)	(0%)	(0%)	(10%)	(12.1%)	(17.4%)	(8%)
Total IgE	341**	81.1	412	272	502	252
GM (95% CI)	(198–587)	(47.0–140)	(252–673)	(150–492)	(271–931)	(146–436)
BMI	18.9†	18.1†	17.2	16.5	16.5	16.7
Mean (95% CI)	(17.6–20.2)	(17.3–18.9)	(16.3–18.1)	(15.9–17.0)	(15.6–17.4)	(15.7–17.6)

** *P*<0.005 for Fisher's exact test between asthmatic and non-asthmatic children within the community.

* *P*<0.05 for Fisher's exact test between asthmatic and non-asthmatic children within the community.

† BMI for children in the UA school for asthma and controls were significantly higher than the other schools, but the two UA groups were not different.

α-gal, galactose-α-1,3-galactose; CI, confidence interval; BMI, body mass index; GM, geometric mean.

#### Titre of immunoglobulin E antibodies

**Fig. 1:**
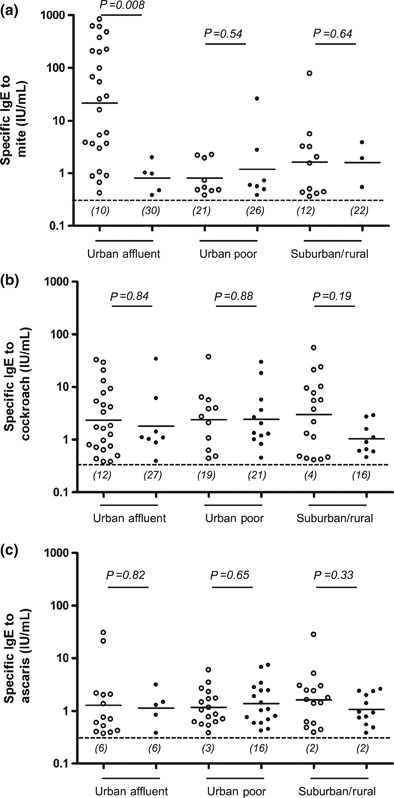
Relationship between the titre of allergen-specific IgE and asthma. Serum obtained from children with asthma (open circles) and without asthma (closed circles) attending the urban affluent, urban poor or suburban/rural schools were assayed for specific IgE to mite (a), cockroach (b) or *Ascaris* (c). The limit of detection for IgE antibodies, represented by the dashed line across the graph, is 0.35 IU/mL. Horizontal lines for each condition indicate the geometric mean of the positive results and values in parenthesis represent the number of samples with negative results. Statistical significance was assessed by a Mann–Whitney *U*-test.

The comparison of the quantitative titre of IgE antibody responses among the positive results showed striking differences between the communities (Fig. 1). Among UP children, there was not only no relationship between the prevalence of sensitization and asthma, but also no difference in IgE titre for any of the allergens. In the SR children, although the prevalence of IgE antibodies to mite and cockroach was higher among those with asthma, there was no significant difference in the titre of IgE antibodies to mite, and only a modest increase in the titre of IgE antibodies to cockroach (Fig. 1). The truly striking finding was that the GM titres of IgE antibodies to mite were highly significantly increased among UA children with asthma (21.2 IU/mL, *n*=25) compared with non-asthmatic controls (0.8 IU/mL, *n*=5) (*P*<0.001). Indeed, 20 (out of 35) of these children had class 3 or higher (≥3.5 IU/mL) IgE antibodies compared with four (out of 53) children with asthma in the other two schools.

### Exercise-induced bronchospasm, immunoglobulin E antibodies and body mass index

**Fig. 2:**
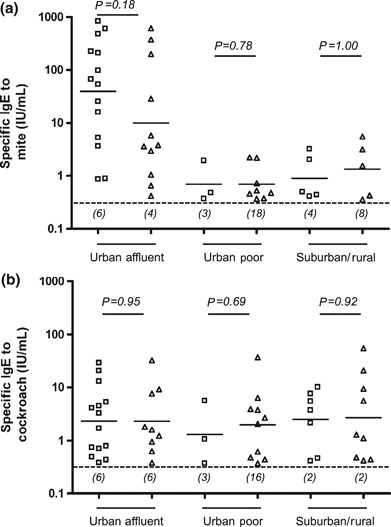
Relationship between the titre of specific IgE to mite, exercise-induced bronchospasm (EIB) and asthma. Serum obtained from asthmatic children with EIB (open squares) and without EIB (open triangles) attending the urban affluent, urban poor or suburban/rural schools were assayed for specific IgE to mite (a) and cockroach (b). The limit of detection for IgE antibodies, represented by the dashed line across the graph, is 0.35 IU/mL. Horizontal lines for each condition indicate the geometric mean of the positive results and values in parenthesis represent the number of samples with negative results. Statistical significance was assessed by a Mann–Whitney *U*-test.

All but two of the children studied had performed exercise challenge test. As expected, there was a very strong association between EIB and asthma. We observed significantly higher prevalence of EIB among cases from UA compared with UP/SR community (20/35 vs. 14/52, respectively, *P*<0.001). We then proceeded to investigate whether there was a difference in sensitization between children who had both asthma and EIB compared with those with asthma but without EIB. Our analysis did not show significant differences in IgE antibody titre between asthmatic children with EIB and without EIB (Fig. 2). Overall, among children with asthma/wheeze, the IgE profile was very similar in those with and those without EIB. In the UA school, both prevalence and titres of IgE antibodies to mite and cockroach did not differ significantly between children with asthma/wheeze who had EIB and those who did not. Similarly, we found no differences in sensitization to cat, dog, *Ascaris* or α-gal (data not shown).

Although BMI was significantly higher among UA children (Table 1), we did not find a significant association between BMI and asthma. Furthermore, in UA asthmatic children, BMI of those with EIB was not different from those without EIB. In contrast, among UP and SR children with asthma/wheeze, those with EIB (*n*=14) had a significantly higher BMI than those without EIB (*n*=38) (18.2 vs. 16.4, respectively, *P*<0.01).

## Discussion

Kumasi has a population of 1 500 000 and is similar to many large towns in sub-Saharan African in that asthma is increasing both in prevalence and severity [10]. The results reported here provide evidence about the differences between children in a relatively affluent urban community and those living in less affluent areas of the same town. The initial publication on these children reported that the prevalence of EIB within the population as a whole had increased from 3.1% to 5.2% over a 10-year period, and that EIB was more common among children attending the urban affluent school (8.3%) as compared with children attending the urban poor (3.0%) or suburban/rural (3.9%) schools [10]. The present results show that there are several features of the children in the urban affluent school that are typical of a westernized community. They had a higher BMI, a lower prevalence of IgE antibodies to *Ascaris*, and no detectable IgE to α-gal. In addition, these children had lower total IgE, high prevalence of IgE to mite, and strikingly higher titres of IgE antibodies to mite. Indeed, the mean titre of IgE antibodies to mite (22 IU/mL) was similar to values reported from New Zealand [21]. The important aspect of our results is that these changes are occurring over a relatively short distance within a town. The implication is that the changes that are necessary to create an increased risk of allergic asthma can occur with only modest changes and over a short period of time.

We acknowledge that matched statistical analysis would be more appropriate based on the study design. However, given the fact that 17 children did not provide blood samples for IgE measurement, a matched analysis would have resulted in exclusion of a total of 34 children (17 pairs). We were keen to include all available data in the analysis, and thus carried out the analysis which did not account for matching, recognising that this may have resulted in the loss of power to detect associations.

Total IgE was significantly lower in the urban affluent compared with children attending the urban poor or suburban/rural school. However, while among urban affluent children, total IgE was significantly higher in those with asthma compared with controls, no such relationship was observed in urban poor or suburban/rural children (Table 3). In addition, the level of mite-specific IgE was a much stronger predictor of asthma than total IgE. This is consistent with previous reports from Kenya which showed that children from an industrialized community not only had an increased prevalence of EIB but also decreased total IgE and increased specific IgE to mite when compared with children from a rural area [4].

Several past studies support an association between asthma severity and the ability to generate a strong IgE antibody response to mite [21, 22]. We found the highest titres of specific IgE to mite in urban affluent children with asthma.

As reported previously, the prevalence of EIB among children with asthma was highest in the urban affluent school. Given the close correlation between asthma diagnosis and observed EIB, as well as the prevalence of IgE antibody to mite in this EIB group, we suggest that EIB can serve as a useful surrogate marker for allergic asthma in this community. However, it should be noted that the prevalence of EIB was not equal to the prevalence of physician-diagnosed asthma within these populations. In keeping with previous findings, we found only 40% of the children with physician-diagnosed asthma exhibited a drop in pulmonary function following exercise [23]. In contrast, all but one child with documented EIB were classified as cases. Such a discrepancy between the two could reflect either an over-diagnosis of asthma in the population or the fact that exercise challenge results are influenced by poor expiratory effort, and can be reduced by environmental factors including air temperature or humidity [16].

Over the last 10 years, there has been a debate concerning the role of helminth infections in the development of wheezing [24]. For example, case studies from Ethiopia suggested that *Ascaris* is protective against wheezing, but reports from Costa Rica and elsewhere suggest that this nematode can enhance asthma and atopic disease [25–28]. Furthermore, a recent study from South Africa indicated that *Ascaris* may have dual effects, being able to both enhance EIB development and reduce sensitization to inhalant allergens [29]. In the present study, in the whole population *Ascaris* sensitization was associated with asthma in the univariate, but not in the multivariate analysis (which could reflect the low worm loads observed). However, in keeping with the socio-economic status of the children examined, sensitization to *Ascaris* was more common in the urban poor and suburban/rural communities where the prevalence of asthma was lower. Taken together, these observations suggest that the IgE antibody response to *Ascaris* is not a major factor driving the development of asthma in this population.

Preliminary evidence from our laboratory suggests an association between having detectable IgE to α-gal and exposure to ectoparasites such as ticks [30]. We also reported a high prevalence of IgE to α-gal among children in a rural village in Kenya [30]. In keeping with that view, the current study found an increased prevalence of IgE to α-gal in children of the urban poor and the suburban/rural communities, who we assume have more environmental exposures. However, this study did not find any association between having detectable IgE to α-gal and asthma.

Children in the urban affluent school had significantly higher BMI, which could reflect less physical activity or different food consumption. While the ‘elevated’ levels of BMI reported here (i.e. 18) are not regarded as obese according to an international standard for childhood obesity [31], the mean BMI of the 14 children with EIB in the two less affluent schools was increased compared with children in the same school with asthma but without EIB. The implication is that the changes in lifestyle occurring within Kumasi are sufficient to influence the prevalence and perhaps the severity of asthma [32, 33].

We assume that the lower levels of IgE antibodies to *Ascaris* and lack of IgE antibodies to α-gal reflect differences in hygiene. In this town, ‘cleanliness’ undoubtedly affects water quality, wearing shoes and treatment with anti-helminthic drugs. Taken together, the results suggest that the changes in diet, exercise and cleanliness that are typical of more affluent areas in African towns are sufficient to produce major changes in asthma over short distances and over a relatively short number of years.

### Clinical relevance

Multiple factors are contributing to the increase in asthma, including but not limited to increased environmental cleanliness, reduced *Ascaris* infections, change in diet and decreased physical activity.
